# Maternal risk factors associated with the birth of preterm infants in the West of Iran: a matched case-control study

**DOI:** 10.1186/s12884-025-07395-5

**Published:** 2025-03-13

**Authors:** Leila Jafarpour, Nasrin Galehdar, Yaser Mokhayeri, Kowsar Qaderi, Mahmood Fakhri

**Affiliations:** 1 Department of Operating Room, School of Paramedical Sciences, Kermanshah University of Medical Sciences, Kermanshah, Iran; 2 Social Determinants of Health Research Center, School of Allied Medical Sciences, Lorestan University of Medical Sciences, Khorramabad, Iran; 3 Cardiovascular Research Center, Shahid Rahimi Hospital, Lorestan University of Medical Sciences, Khorramabad, Iran; 4 Assistant Professor Reproductive Health, Clinical Research Development Center, Motazedi Hospital, Kermanshah University of Medical Sciences, Kermanshah, Iran; 5 Faculty of Paramedical, Kermanshah University of Medical Sciences, Kermanshah, Iran

**Keywords:** Infant, Premature, Premature birth, Risk factors

## Abstract

**Background:**

Preterm birth is one of the global most common causes of mortality among infants, especially in developing countries. Therefore, the present study was conducted to determine the maternal risk factors related to the birth of preterm infants.

**Methods:**

The present case-control study was conducted on 220 premature infants as the case group and 440 term infants as the control group in the hospitals of Khorram Abad, Iran, in 2023. Two groups were matched in terms of gender and date of birth. Data were gathered by the researcher using a researcher-made questionnaire, interviewing the mothers and physicians, and reviewing mothers’ medical files in maternity and postpartum wards. Data were analyzed using Stata 17 software and descriptive statistics and conditional logistic regression test at the significance level of less than 0.05.

**Results:**

In multivariable analysis adjusted odds ratio (AOR) was estimated, mother’s employment (AOR: 2.85; 95%CI:1.05–7.77), history of abortion (AOR: 2.04; 95%CI: 1.10–3.78), sexual activity from 32nd to 36.6th week of pregnancy (AOR: 0.33; 95%CI: 0.20–0.54), pre-eclampsia (AOR: 11.09; 95%CI: 4.5-27.39), premature rupture of membrane (AOR: 6.76; 95%CI; 3.7-12.34) and placental abruption (AOR: 16.07; 95%CI: 5.45–47.39) were significantly associated with preterm birth of infants. No significant relation was observed between mother’s age, assisted reproductive treatment, cervical insufficiency, and the number of received prenatal cares at the health centers and the birth of premature infants.

**Conclusion:**

According to the results of the study, factors such as a mother’s employment, history of abortion, pre-eclampsia, premature rupture of membranes, and placental abruption can affect the birth of premature infants. Therefore, the control of maternal factors influential in the birth of premature infants, as well as care during pregnancy, can reduce the occurrence of premature births, followed by the reduction of healthcare costs and infant mortality and the improvement of the youth level of the population.

**Supplementary Information:**

The online version contains supplementary material available at 10.1186/s12884-025-07395-5.

## Background

Infant mortality is one of any country’s most important health indices [1]. According to the reports by the World Health Organization, annually, about 15 million preterm births occur in the world. About 90% of the preterm deliveries happen in developing countries, and 85% of them belongs to Africa and Asia [2, 3]. Preterm infants are those who are born before the 37th week of pregnancy [4]. According to the definition by the World Health Organization, being preterm is categorized into three divisions: extremely preterm (less than 28 weeks), very preterm (28 to less than 32 weeks), and moderate to late preterm (32 to less than 37 weeks) [5]. Although modern methods of care for preterm infants have improved the condition of these little creatures to some extent, but no stable significant decrease has not occurred in the rate of preterm birth of infants [6]. The prevalence of premature delivery in the United States was 9.93% in 2017 and was increased to 10.38% in 2022 [7]. According to the results of a meta-analysis, the prevalence of premature delivery in Iran was 9.2% [8]. Based on the statistical reports by the World Health Organization, about 60–80% of infant mortalities that are not associated with congenital anomalies, are caused by preterm birth [9]. Also, preterm birth was the second most common cause of death among children of under 5, after pneumonia [1]. During the past 20 years, two third of the mortalities during the first 24 h after birth was among preterm infants in Iran [10]. A wide range of maternal, fetal and placental factors are effective in preterm birth of infants [11]. These factors include infection, bleeding, uterus and cervix related factors, oligohydramnios, polyhydramnios, diabetes mellitus, gestational hypertension, multiple birth, short interval between pregnancies, history of abortion, preterm premature rupture of membranes, low body mass index before pregnancy, obesity and demographic factors [3, 12, 13]. In the study of XU et al. [14] in China, the high age of the mother, low education level of the mother, use of assisted reproduction treatments, urban residence, and male gender of the infant were associated with premature birth.

The study conducted by Ahumada-Barrios et al. [15] in Peru showed that there is a significant relationship between twin pregnancy, inadequate care during pregnancy, lack of care during pregnancy, history of preterm birth, and pre-eclampsia with the birth of a premature infant.

In a study conducted by Hidayat et al. [16], maternal age below 20 years, prenatal bleeding, and anemia were associated with preterm delivery.

Preterm infants are exposed to various complications such as necrotizing enterocolitis, patent ductus arteriosus, intraventricular hemorrhage, and sepsis, and also risks such as cerebral palsy, mental retardation, cognitive, respiratory, hearing, and vision problems, and increased risk for diseases such as hypertension and diabetes in adulthood. The economic cost of the birth of a preterm infant has been reported as 10 times more than a term infant [17–19]. However, differences in social factors in different societies such as the age of marriage and pregnancy and genetic, anatomic and physiologic differences in human populations would magnify the necessity for performing more extended studies with acceptable sample sizes. Therefore, the present study was conducted to determine the maternal risk factors associated with the birth of preterm infants in the hospitals of Khorram Abad, so that by identifying these factors, an effective step could be taken toward preventing the birth of preterm infants and death and disability related to this complication.

## Methods

The present case-control study was conducted to determine the maternal risk factors associated with the birth of preterm infants in the hospitals of Khorram Abad, Iran, from March to September 2023. Study population included all the infants who were born during the study period (term and preterm) in the hospitals of Khorram Abad. The sample size for this study was calculated to be 660 by calculating the minimum required samples (220 cases and 440 controls) who were selected and enrolled in the study using convenience sampling method. The case group included 220 preterm infants who were just born during the time of the study and for each preterm infant, 2 term infants who were born right after term were selected for the control group. For the case group, infants with gestational age of less than 37 weeks and for the control group, infants with gestational age of 37 to 42 weeks were selected. Infants with congenital and genetic anomalies and stillbirths were excluded from the study. To achieve more accurate data for determining the cause of preterm birth, some variables such as gender and birthdate were matched between the study groups. Gestational age was determined based on the first day of the last menstruation or the ultrasound of the first three months. Data was gathered using a researcher-made questionnaire including mother’s age, mother’s employment, history of abortion, assisted reproductive treatment, cervical insufficiency, sexual activity from the 32nd to the 36.6th of pregnancy, number of received prenatal cares at health centers, pre-eclampsia, premature rupture of membrane and placental abruption. Questionnaires were completed by the researcher by interviewing the mothers and physicians and reviewing mothers’ medical files at the maternity and postpartum wards. Statistical analysis was done using Stata 17 software. To compare the risk factors in both groups, conditional logistic regression test was performed to calculate odds ratio in univariate and multivariable models and for describing the data, descriptive statistics was used. The level of significance was set at *p* < 0.05 for all the statistical tests.

## Results

Most mothers were in the age group of 20 to 35 years and were not employed. More than two-thirds of the mothers had no history of abortion. Most of the mothers were visited by health centers more than four times. More than 90% of the case and control groups had not used assisted reproductive treatments, and nearly one-third of the case group and more than half of the control group had sexual activity from the 32nd to 36.6th week of pregnancy. Other characteristics of mothers are shown in Table 1. According to the results of multivariable analysis, the Adjusted odds ratio (AOR) of preterm birth among employed mothers was 2.85 times more than housewife mothers (AOR: 2.85; 95%CI: 1.05–7.77), in mothers with history of abortion was 2.04 times more than mothers without history of abortion (AOR: 2.04; 95%CI: 1.10–3.78), in mothers with pre-eclampsia was 11.09 times more than healthy mothers (AOR: 11.09; 95%CI: 4.5-27.39), in mothers with premature rupture of membrane was 6.76 times more than normal mothers (AOR: 6.76; 95%CI: 3.7-12.34), and in mothers with placental abruption was 16.07 times more than mothers without placental abruption (AOR: 16.07; 95%CI: 5.45–47.39). Mothers who had sexual activity from the 32nd to 36.6th week of pregnancy had a 0.33 lower chance of giving birth to preterm infants (AOR: 0.33; 95%CI: 0.20–0.54). No significant relation was observed between mother’s age, assisted reproductive treatment, cervical insufficiency and the number of received prenatal cares at health centers and the odds for preterm birth of infants (Table 2).

**Table 1:** Frequency distribution of the studied variables in preterm and term groups

Variable		Preterm (case)	Term (control)	Total
		No. (percent)	No. (percent)	No. (percent)
Mother’s age	Under 20 years	14 (6.36)	40 (9.09)	54 (7.72)
	20–35 years	143 (65)	313 (71.14)	456 (68.07)
	Over 35 years	63 (28.64)	87 (19.77)	150 (24.20)
Mother’s employment	Yes	23 (10.45)	18 (4.09)	41 (7.27)
	No	197 (89.55)	422 (95.91)	619 (92.73)
History of abortion	Yes	72 (32.73)	104 (23.64)	176 (28.18)
	No	148 (67.27)	336 (76.36)	484 (71.81)
assisted reproductive treatment	Yes	25 (11.36)	19 (4.32)	44 (7.84)
	No	195 (88.64)	421 (95.68)	616 (92.16)
Cervical insufficiency	Yes	8 (3.64)	5 (1.14)	13 (2.39)
	No	212 (96.36)	435 (98.86)	647 (97.61)
Sexual activity from 32nd to 36.6th week of pregnancy	Yes	67 (30.45)	263 (59.77)	330 (45.11)
	No	153 (69.55)	177 (40.23)	330 (54.89)
Number of received prenatal cares at health centers	0 times	11 (5)	7 (1.59)	18 (3.29)
	1–2 times	14 (6.36)	15 (3.41)	29 (4.88)
	3–4 times	18 (8.18)	27 (6.14)	45 (7.16)
	More than 4 times	177 (80.45)	391 (88.86)	568 (84.65)
Pre-eclampsia	Yes	39 (17.73)	14 (3.18)	53 (10.45)
	No	181 (82.27)	426 (96.82)	607 (89.54)
premature rupture of membrane	Yes	76 (34.55)	66 (15)	142 (24.77)
	No	144 (65.45)	374 (85)	518 (75.22)
Placental abruption	Yes	21 (9.55)	9 (2.05)	30 (5.8)
	No	199 (90.45)	431 (97.95)	630 (94.2)

**Table 2:** Univariate and multivariable logistic regression assessing preterm birth of infants

Variables		Univariate analysis			Multivariable analysis		
		Odds ratio	*P*- value	95% confidence interval	Adjusted Odds ratio	*P*- value	95% confidence interval
Mother’s age	Under 20 years	1 (reference)	-	-	1 (reference)	-	-
	20–35 years	1.32	0.37	0.71–2.46	1.70	0.25	0.68–4.21
	Over 35 years	2.05	0.03	1.03–4.05	2.75	0.07	0.91–8.25
Mother’s employment	NO	1 (reference)	-	-	1 (reference)	-	-
	Yes	2.55	0.003	1.37–4.73	2.85	0.04	1.05–7.77
History of abortion	NO	1 (reference)	-	-	1 (reference)	-	-
	Yes	1.54	0.01	1.08–2.18	2.04	0.02	1.10–3.78
assisted reproductive treatment	NO	1 (reference)	-	-	1 (reference)	-	-
	Yes	3.11	0.001	1.6–6.04	2.46	0.05	0.99–6.11
Cervical insufficiency	NO	1 (reference)	-	-	1 (reference)	-	-
	Yes	3.2	0.04	1.04–9.78	1.67	0.47	0.4–6.89
Sexual activity from 32nd to 36.6th week of pregnancy	NO	1 (reference)	-	-	1 (reference)	-	-
	yes	0.28	< 0.001	0.19–0.40	0.33	< 0.001	0.20–0.54
Number of received prenatal cares at health centers	0 times	1 (reference)	-	-	1 (reference)	-	-
	1–2 times	0.60	0.41	0.18–2.01	1. 40	0.7	0.25–7.86
	3–4 times	0.45	0.16	0.14–1.37	0.69	0.66	0.13–3.53
	More than 4 times	0.31	0.01	0.12–0.80	0.48	0.3	0.12–1.93
Pre-eclampsia	NO	1 (reference)	-	-	1 (reference)	-	-
	Yes	6.26	< 0.001	3.27–12	11.09	< 0.001	4.5–27.39
Premature rupture of membrane	NO	1 (reference)	-	-	1 (reference)	-	-
	Yes	3.22	< 0.001	2.13–4.87	6.76	< 0.001	3.7–12.34
Placental abruption	NO	1 (reference)	-	-	1 (reference)	-	-
	Yes	4.66	< 0.001	2.13–10.19	16.07	< 0.001	5.45–47.39

## Discussion

Preterm birth of infant is one of the serious obstetric complications and it has various related factors. In the present study, the odds of preterm birth of infant were 2.85 times higher in employed mothers compared to housewife mothers. In a study by Purwanderi et al. [20] the odds of late preterm birth of infants was 16.9 times higher among employed mothers than housewife mothers. Also, the study of Stylianou-Riga et al. [21] in Cyprus showed that long working hours would increase the chance of premature labor about 4 more times. These results were in line with the results of the present study. The study by Nwoga et al. [22] in Nigeria showed that mother’s occupation had no significant effect on gestational age at the time of delivery and infant’s birth weight. In the present study, the odds of preterm birth of infant was 2.04 times higher in mothers who had a history of abortion; this result was in line with the results of some previous studies including the study by Wakeyo et al. [23–25]. A study by Ke et al. [26] in southern China showed that in first-time mothers, previous induced abortion was not associated with an increased risk of preterm birth in the next pregnancy. The results of this study were inconsistent with the present study. The relation between history of abortion and premature labor might be due to the performed procedures and aggressive measures that have been conducted for management of abortion, which might lead to cervical insufficiency and consequently, premature labor in next pregnancies [27]. Results of the present study showed that sexual activity from 32nd to 36.6th week of pregnancy would decrease the risk of preterm birth of infants. The study by Trivedi et al. [28] showed that women who had sexual activity during pregnancy had 3 times higher chance of premature labor. The study of Kong et al. [29]showed no significant relation between sexual activity, its number and time and obstetric and neonatal outcomes. It seems that, the reason for different results in the present study was the defined time period for sexual activity in the present study. One of the effective factors on preterm birth of infants is pre-eclampsia; similar to other studies, the odds for preterm birth of infants among mothers with pre-eclampsia was 11.09 times more than other mothers [15, 30, 31]. In the study by khalajinia et al. [32], no significant relation was observed between preterm birth of infants and pre-eclampsia. Hypertension would decrease uteroplacental blood flow, therefore, it would restrict intrauterine fetal growth and so, would lead to premature labor [33]. In the present study, the odds for preterm birth of infants in mothers with premature rupture of membrane was 6.76 times higher than other mothers; this result was in line with the results of the studies by Dahman [34], Sifer [35] and Rutayisire [36]. No significant relation was observed between premature rupture of membrane and preterm birth of infants in the study by Carter et al. [37]. The most important cause of premature labor has been mentioned as premature rupture of membrane [38]. Rupture of membrane would lead to more intrauterine infections, more secretion of oxytocin and consequently, induction of labor which could cause premature labor [6]. Placental abruption before term delivery would lead to vaginal bleeding, hemorrhagic shock and fetal mortality and could lead to emergency delivery before due [39]. In the present study the odds for preterm birth of infants in mothers with placental abruption was 16.07 times higher than other mothers. In the study by Temu et al. [39] the chance of preterm birth of infants in mothers with placental abruption was 5.4 times higher than other mothers, which was in line with the results of the present study.

## Conclusion

In the present study, a significant relation was observed between preterm birth of infants and mother’s employment, history of abortion, sexual activity from 32nd and 36.6th week of pregnancy, pre-eclampsia, premature rupture of membrane and placental abruption. Therefore, considering that some of the causes of preterm birth of infants are preventable, recognizing at risk mothers and performing prenatal cares could decrease the rate of preterm birth and infant mortality and also, help population rejuvenation, which is one of the goals of the Ministry of Health and Medical Sciences of Iran. In this research, limited factors have been examined, so it is suggested that other factors be reviewed on a wider level and with a larger number of samples. It is also recommended that a study be conducted on these infants’ health status and quality of life in their young years.

## Limitation

In this research, a questionnaire was used to collect data, as a result, some people may have refused to provide accurate answers and gave unrealistic answers.

## Electronic supplementary material

Below is the link to the electronic supplementary material.

Supplementary Material 1

## Data Availability

Data sets collected and analyzed during the current study are available from the corresponding author upon reasonable request.
