# Supplementary material for: Maternal risk factors associated with the birth of preterm infants in the West of Iran: a matched case-control study
Source: BMC Pregnancy Childbirth. 2025 Mar 13;25:278. doi: 10.1186/s12884-025-07395-5 (PMC11905591; doi:10.1186/s12884-025-07395-5)
Supplement: Supplementary file 1 — Supplementary Material 1 [file 12884_2025_7395_MOESM1_ESM.docx]

questionnaire

| Mother's age | .1Under 20 years .2 20-35 years .3 Over 35 years |
| --- | --- |
| Mother's employment | .1Yes .2 No |
| History of abortion | .1Yes .2 No |
| assisted reproductive treatment | .1Yes .2 No |
| Cervical insufficiency | .1Yes .2 No |
| Sexual activity from 32^nd^ to 36.6^th^ week of pregnancy | .1Yes .2 No |
| Number of received prenatal cares at health centers | 1. 0 times 2. 1-2 times 3. 3-4 times 4. More than 4 times |
| Preeclampsia | .1Yes .2 No |
| premature rupture of membrane | .1Yes .2 No |
| Placental abruption | .1Yes .2 No |
